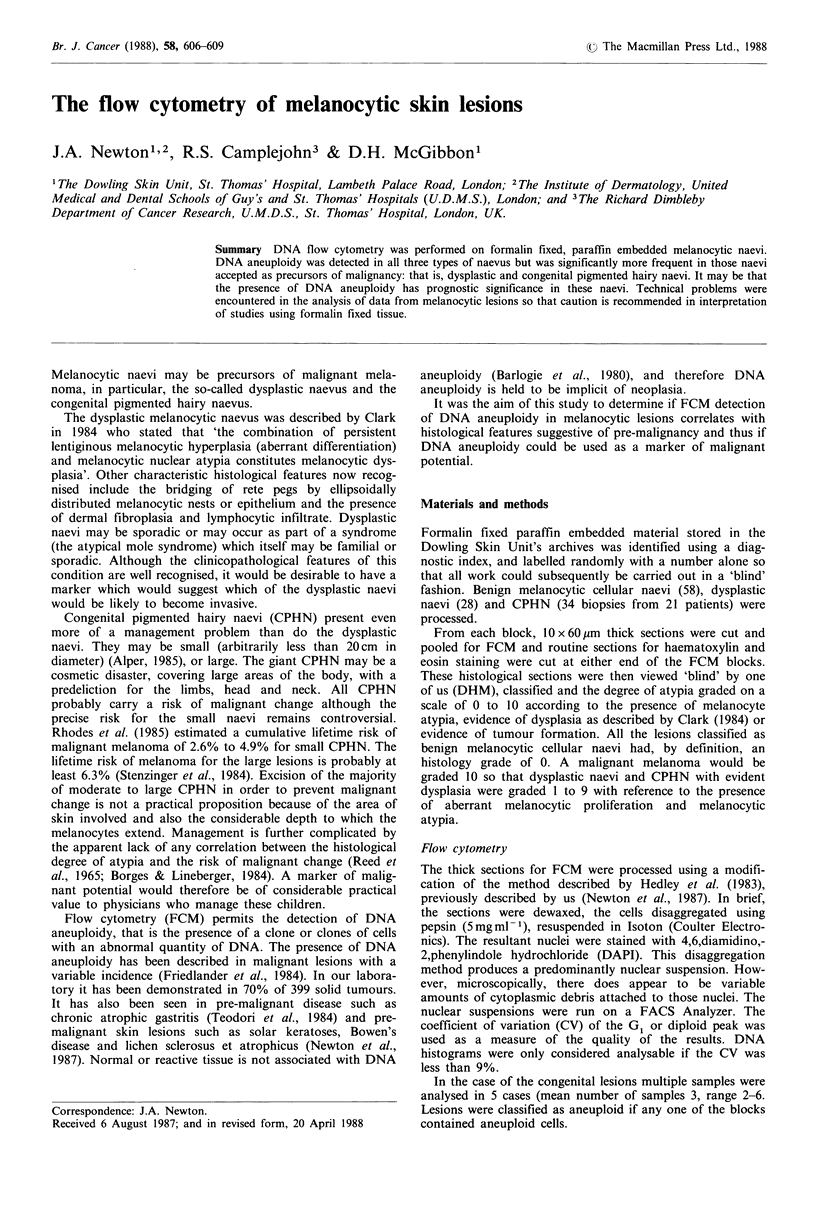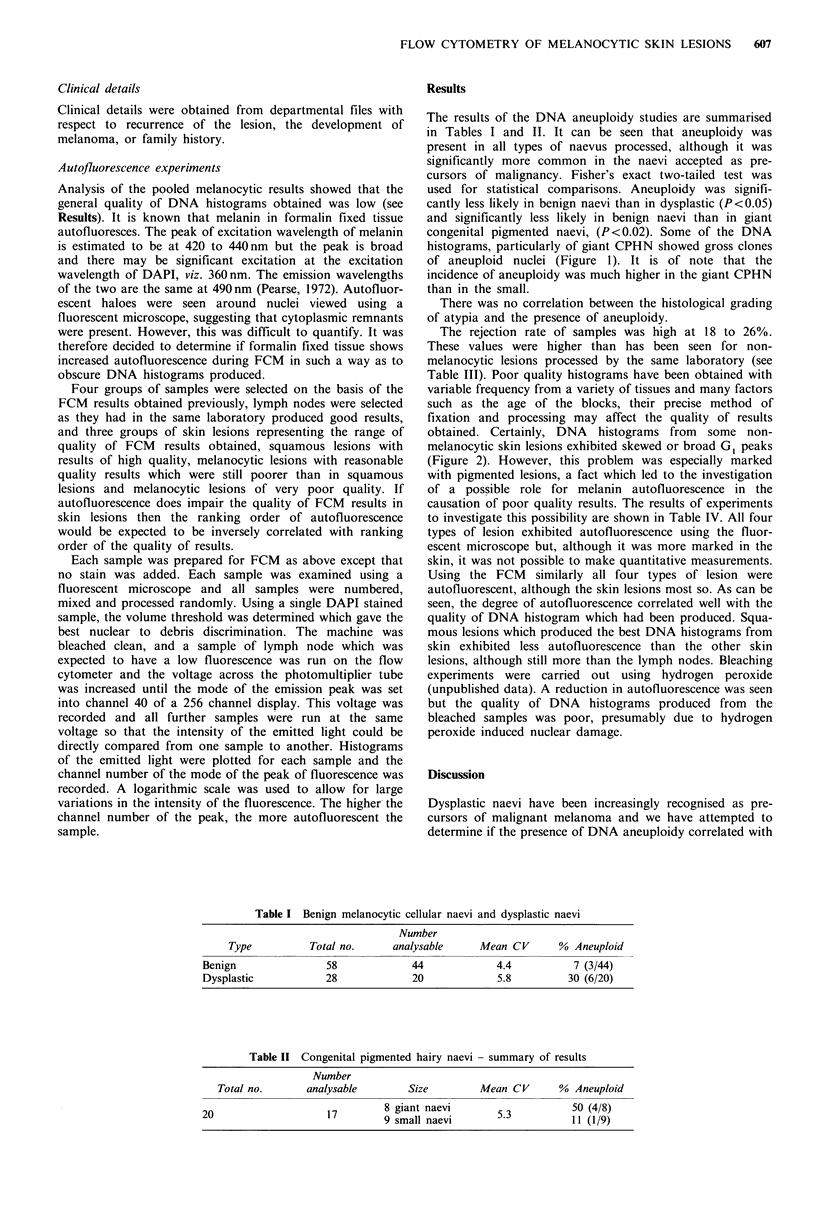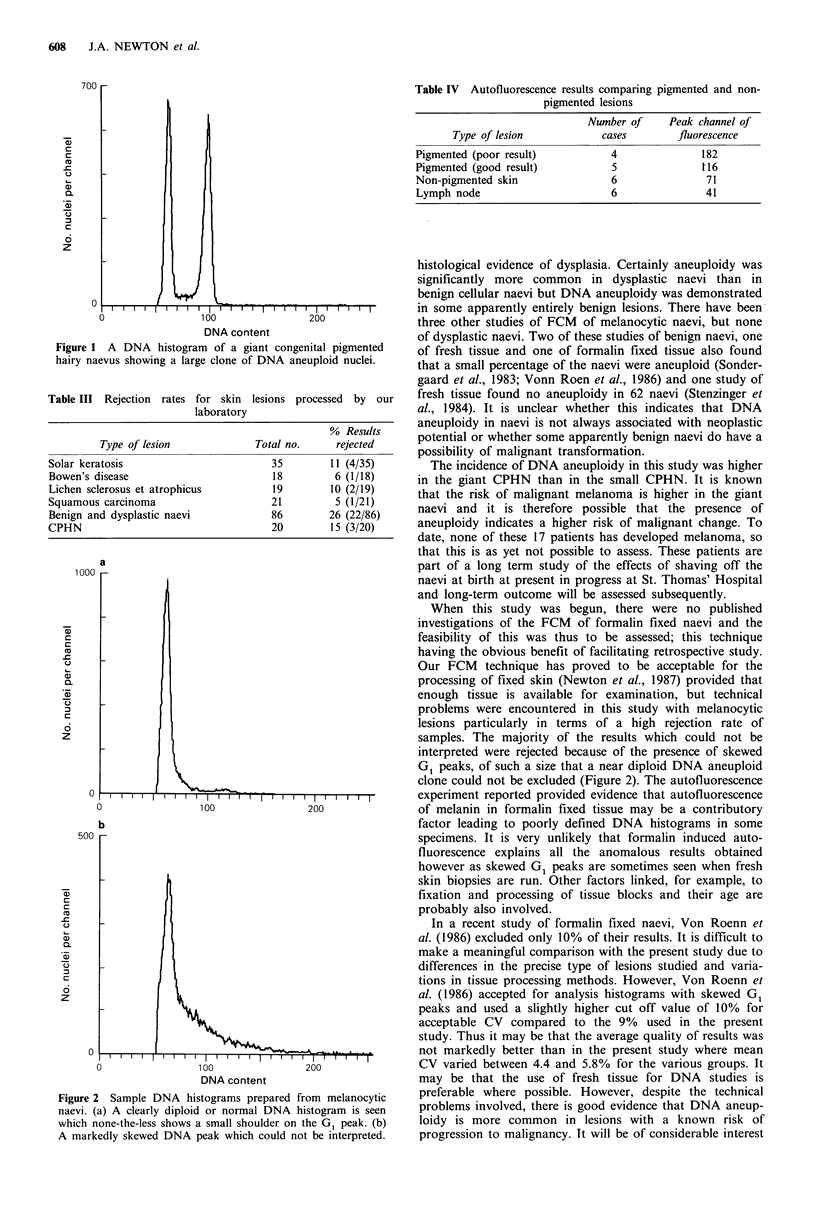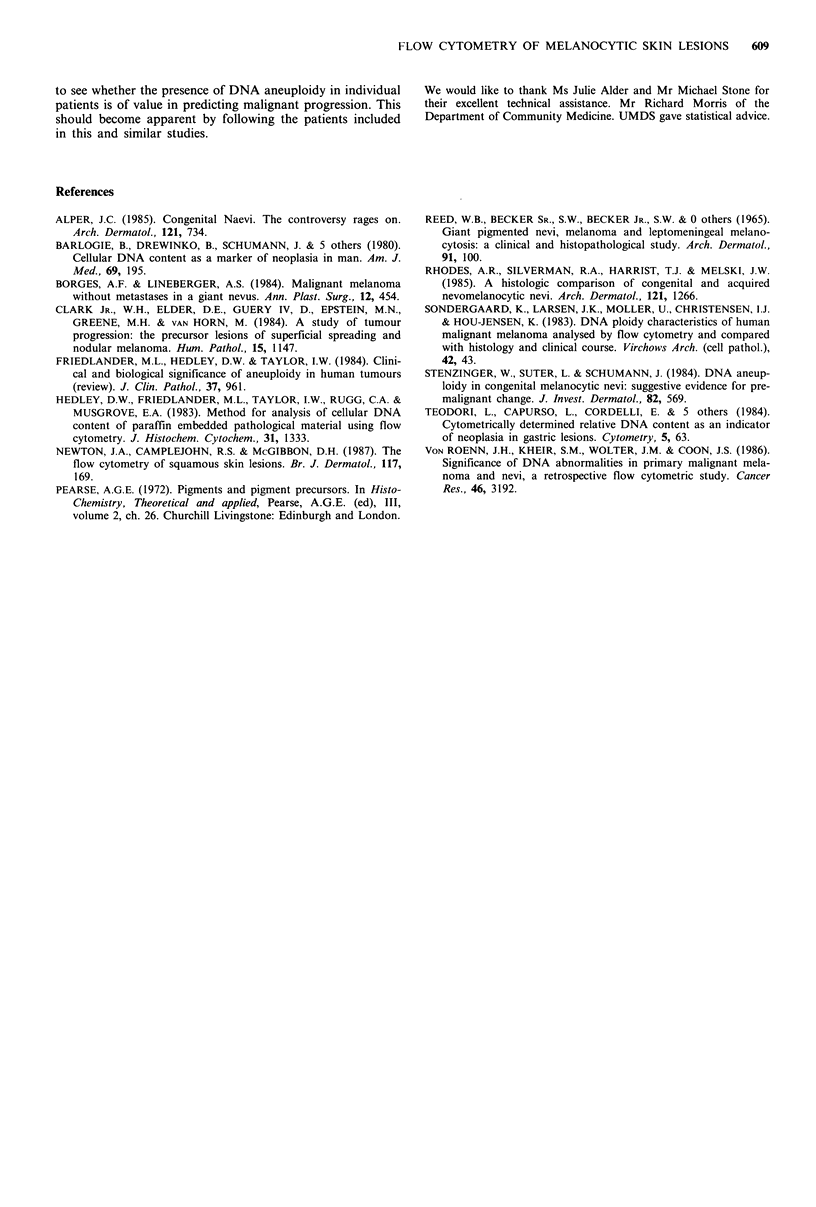# The flow cytometry of melanocytic skin lesions.

**DOI:** 10.1038/bjc.1988.268

**Published:** 1988-11

**Authors:** J. A. Newton, R. S. Camplejohn, D. H. McGibbon

**Affiliations:** Dowling Skin Unit, St. Thomas' Hospital, London, UK.

## Abstract

DNA flow cytometry was performed on formalin fixed, paraffin embedded melanocytic naevi. DNA aneuploidy was detected in all three types of naevus but was significantly more frequent in those naevi accepted as precursors of malignancy: that is, dysplastic and congenital pigmented hairy naevi. It may be that the presence of DNA aneuploidy has prognostic significance in these naevi. Technical problems were encountered in the analysis of data from melanocytic lesions so that caution is recommended in interpretation of studies using formalin fixed tissue.


Br. J. Cancer (1988), 58, 606–609 © The Macmillan Press Ltd., 1988

### The flow cytometry of melanocytic skin lesions

J.A. Newton[1,2], R.S. Camplejohn[3] & D.H. McGibbon[1]

[1]*The Dowling Skin Unit, St. Thomas' Hospital, Lambeth Palace Road, London;* [2]*The Institute of Dermatology, United Medical and Dental Schools of Guy's and St. Thomas' Hospitals (U.D.M.S.), London; and* [3]*The Richard Dimbleby Department of Cancer Research, U.M.D.S., St. Thomas' Hospital, London, UK.*

**Summary** DNA flow cytometry was performed on formalin fixed, paraffin embedded melanocytic naevi. DNA aneuploidy was detected in all three types of naevus but was significantly more frequent in those naevi accepted as precursors of malignancy: that is, dysplastic and congenital pigmented hairy naevi. It may be that the presence of DNA aneuploidy has prognostic significance in these naevi. Technical problems were encountered in the analysis of data from melanocytic lesions so that caution is recommended in interpretation of studies using formalin fixed tissue.

Melanocytic naevi may be precursors of malignant melanoma, in particular, the so-called dysplastic naevus and the congenital pigmented hairy naevus.

The dysplastic melanocytic naevus was described by Clark in 1984 who stated that 'the combination of persistent lentiginous melanocytic hyperplasia (aberrant differentiation) and melanocytic nuclear atypia constitutes melanocytic dysplasia'. Other characteristic histological features now recognised include the bridging of rete pegs by ellipsoidally distributed melanocytic nests or epithelium and the presence of dermal fibroplasia and lymphocytic infiltrate. Dysplastic naevi may be sporadic or may occur as part of a syndrome (the atypical mole syndrome) which itself may be familial or sporadic. Although the clinicopathological features of this condition are well recognised, it would be desirable to have a marker which would suggest which of the dysplastic naevi would be likely to become invasive.

Congenital pigmented hairy naevi (CPHN) present even more of a management problem than do the dysplastic naevi. They may be small (arbitrarily less than 20 cm in diameter) (Alper, 1985), or large. The giant CPHN may be a cosmetic disaster, covering large areas of the body, with a predeliction for the limbs, head and neck. All CPHN probably carry a risk of malignant change although the precise risk for the small naevi remains controversial. Rhodes *et al.* (1985) estimated a cumulative lifetime risk of malignant melanoma of 2.6% to 4.9% for small CPHN. The lifetime risk of melanoma for the large lesions is probably at least 6.3% (Stenzinger *et al.*, 1984). Excision of the majority of moderate to large CPHN in order to prevent malignant change is not a practical proposition because of the area of skin involved and also the considerable depth to which the melanocytes extend. Management is further complicated by the apparent lack of any correlation between the histological degree of atypia and the risk of malignant change (Reed *et al.*, 1965; Borges & Lineberger, 1984). A marker of malignant potential would therefore be of considerable practical value to physicians who manage these children.

Flow cytometry (FCM) permits the detection of DNA aneuploidy, that is the presence of a clone or clones of cells with an abnormal quantity of DNA. The presence of DNA aneuploidy has been described in malignant lesions with a variable incidence (Friedlander *et al.*, 1984). In our laboratory it has been demonstrated in 70% of 399 solid tumours. It has also been seen in pre-malignant disease such as chronic atrophic gastritis (Teodori *et al.*, 1984) and pre-malignant skin lesions such as solar keratoses, Bowen's disease and lichen sclerosus et atrophicus (Newton *et al.*, 1987). Normal or reactive tissue is not associated with DNA aneuploidy (Barlogie *et al.*, 1980), and therefore DNA aneuploidy is held to be implicit of neoplasia.

It was the aim of this study to determine if FCM detection of DNA aneuploidy in melanocytic lesions correlates with histological features suggestive of pre-malignancy and thus if DNA aneuploidy could be used as a marker of malignant potential.

### Materials and methods

Formalin fixed paraffin embedded material stored in the Dowling Skin Unit's archives was identified using a diagnostic index, and labelled randomly with a number alone so that all work could subsequently be carried out in a 'blind' fashion. Benign melanocytic cellular naevi (58), dysplastic naevi (28) and CPHN (34 biopsies from 21 patients) were processed.

From each block, $10 \times 60\,\mu m$ thick sections were cut and pooled for FCM and routine sections for haematoxylin and eosin staining were cut at either end of the FCM blocks. These histological sections were then viewed 'blind' by one of us (DHM), classified and the degree of atypia graded on a scale of 0 to 10 according to the presence of melanocyte atypia, evidence of dysplasia as described by Clark (1984) or evidence of tumour formation. All the lesions classified as benign melanocytic cellular naevi had, by definition, an histology grade of 0. A malignant melanoma would be graded 10 so that dysplastic naevi and CPHN with evident dysplasia were graded 1 to 9 with reference to the presence of aberrant melanocytic proliferation and melanocytic atypia.

### *Flow cytometry*

The thick sections for FCM were processed using a modification of the method described by Hedley *et al.* (1983), previously described by us (Newton *et al.*, 1987). In brief, the sections were dewaxed, the cells disaggregated using pepsin ($5\,mg\,ml^{-1}$), resuspended in Isoton (Coulter Electronics). The resultant nuclei were stained with 4,6,diamidino,-2,phenylindole hydrochloride (DAPI). This disaggregation method produces a predominantly nuclear suspension. However, microscopically, there does appear to be variable amounts of cytoplasmic debris attached to those nuclei. The nuclear suspensions were run on a FACS Analyzer. The coefficient of variation (CV) of the $G_1$ or diploid peak was used as a measure of the quality of the results. DNA histograms were only considered analysable if the CV was less than 9%.

In the case of the congenital lesions multiple samples were analysed in 5 cases (mean number of samples 3, range 2–6. Lesions were classified as aneuploid if any one of the blocks contained aneuploid cells.

Correspondence: J.A. Newton.
Received 6 August 1987; and in revised form, 20 April 1988

### *Clinical details*

Clinical details were obtained from departmental files with respect to recurrence of the lesion, the development of melanoma, or family history.

### *Autofluorescence experiments*

Analysis of the pooled melanocytic results showed that the general quality of DNA histograms obtained was low (see **Results**). It is known that melanin in formalin fixed tissue autofluoresces. The peak of excitation wavelength of melanin is estimated to be at 420 to 440 nm but the peak is broad and there may be significant excitation at the excitation wavelength of DAPI, *viz.* 360 nm. The emission wavelengths of the two are the same at 490 nm (Pearse, 1972). Autofluorescent haloes were seen around nuclei viewed using a fluorescent microscope, suggesting that cytoplasmic remnants were present. However, this was difficult to quantify. It was therefore decided to determine if formalin fixed tissue shows increased autofluorescence during FCM in such a way as to obscure DNA histograms produced.

Four groups of samples were selected on the basis of the FCM results obtained previously, lymph nodes were selected as they had in the same laboratory produced good results, and three groups of skin lesions representing the range of quality of FCM results obtained, squamous lesions with results of high quality, melanocytic lesions with reasonable quality results which were still poorer than in squamous lesions and melanocytic lesions of very poor quality. If autofluorescence does impair the quality of FCM results in skin lesions then the ranking order of autofluorescence would be expected to be inversely correlated with ranking order of the quality of results.

Each sample was prepared for FCM as above except that no stain was added. Each sample was examined using a fluorescent microscope and all samples were numbered, mixed and processed randomly. Using a single DAPI stained sample, the volume threshold was determined which gave the best nuclear to debris discrimination. The machine was bleached clean, and a sample of lymph node which was expected to have a low fluorescence was run on the flow cytometer and the voltage across the photomultiplier tube was increased until the mode of the emission peak was set into channel 40 of a 256 channel display. This voltage was recorded and all further samples were run at the same voltage so that the intensity of the emitted light could be directly compared from one sample to another. Histograms of the emitted light were plotted for each sample and the channel number of the mode of the peak of fluorescence was recorded. A logarithmic scale was used to allow for large variations in the intensity of the fluorescence. The higher the channel number of the peak, the more autofluorescent the sample.

### Results

The results of the DNA aneuploidy studies are summarised in Tables I and II. It can be seen that aneuploidy was present in all types of naevus processed, although it was significantly more common in the naevi accepted as precursors of malignancy. Fisher's exact two-tailed test was used for statistical comparisons. Aneuploidy was significantly less likely in benign naevi than in dysplastic ($P < 0.05$) and significantly less likely in benign naevi than in giant congenital pigmented naevi, ($P < 0.02$). Some of the DNA histograms, particularly of giant CPHN showed gross clones of aneuploid nuclei (Figure 1). It is of note that the incidence of aneuploidy was much higher in the giant CPHN than in the small.

There was no correlation between the histological grading of atypia and the presence of aneuploidy.

The rejection rate of samples was high at 18 to 26%. These values were higher than has been seen for non-melanocytic lesions processed by the same laboratory (see Table III). Poor quality histograms have been obtained with variable frequency from a variety of tissues and many factors such as the age of the blocks, their precise method of fixation and processing may affect the quality of results obtained. Certainly, DNA histograms from some non-melanocytic skin lesions exhibited skewed or broad $G_1$ peaks (Figure 2). However, this problem was especially marked with pigmented lesions, a fact which led to the investigation of a possible role for melanin autofluorescence in the causation of poor quality results. The results of experiments to investigate this possibility are shown in Table IV. All four types of lesion exhibited autofluorescence using the fluorescent microscope but, although it was more marked in the skin, it was not possible to make quantitative measurements. Using the FCM similarly all four types of lesion were autofluorescent, although the skin lesions most so. As can be seen, the degree of autofluorescence correlated well with the quality of DNA histogram which had been produced. Squamous lesions which produced the best DNA histograms from skin exhibited less autofluorescence than the other skin lesions, although still more than the lymph nodes. Bleaching experiments were carried out using hydrogen peroxide (unpublished data). A reduction in autofluorescence was seen but the quality of DNA histograms produced from the bleached samples was poor, presumably due to hydrogen peroxide induced nuclear damage.

### Discussion

Dysplastic naevi have been increasingly recognised as precursors of malignant melanoma and we have attempted to determine if the presence of DNA aneuploidy correlated with

**Table I** Benign melanocytic cellular naevi and dysplastic naevi

| *Type* | *Total no.* | *Number analysable* | *Mean CV* | *% Aneuploid* |
|---|---|---|---|---|
| Benign | 58 | 44 | 4.4 | 7 (3/44) |
| Dysplastic | 28 | 20 | 5.8 | 30 (6/20) |

**Table II** Congenital pigmented hairy naevi – summary of results

| *Total no.* | *Number analysable* | *Size* | *Mean CV* | *% Aneuploid* |
|---|---|---|---|---|
| 20 | 17 | 8 giant naevi<br>9 small naevi | 5.3 | 50 (4/8)<br>11 (1/9) |

**Figure 1** A DNA histogram of a giant congenital pigmented hairy naevus showing a large clone of DNA aneuploid nuclei.

**Table III** Rejection rates for skin lesions processed by our laboratory

| *Type of lesion* | *Total no.* | *% Results rejected* |
|---|---|---|
| Solar keratosis | 35 | 11 (4/35) |
| Bowen's disease | 18 | 6 (1/18) |
| Lichen sclerosus et atrophicus | 19 | 10 (2/19) |
| Squamous carcinoma | 21 | 5 (1/21) |
| Benign and dysplastic naevi | 86 | 26 (22/86) |
| CPHN | 20 | 15 (3/20) |

**Figure 2** Sample DNA histograms prepared from melanocytic naevi. (a) A clearly diploid or normal DNA histogram is seen which none-the-less shows a small shoulder on the $G_1$ peak. (b) A markedly skewed DNA peak which could not be interpreted.

**Table IV** Autofluorescence results comparing pigmented and non-pigmented lesions

| *Type of lesion* | *Number of cases* | *Peak channel of fluorescence* |
|---|---|---|
| Pigmented (poor result) | 4 | 182 |
| Pigmented (good result) | 5 | 116 |
| Non-pigmented skin | 6 | 71 |
| Lymph node | 6 | 41 |

histological evidence of dysplasia. Certainly aneuploidy was significantly more common in dysplastic naevi than in benign cellular naevi but DNA aneuploidy was demonstrated in some apparently entirely benign lesions. There have been three other studies of FCM of melanocytic naevi, but none of dysplastic naevi. Two of these studies of benign naevi, one of fresh tissue and one of formalin fixed tissue also found that a small percentage of the naevi were aneuploid (Sondergaard *et al.*, 1983; Vonn Roen *et al.*, 1986) and one study of fresh tissue found no aneuploidy in 62 naevi (Stenzinger *et al.*, 1984). It is unclear whether this indicates that DNA aneuploidy in naevi is not always associated with neoplastic potential or whether some apparently benign naevi do have a possibility of malignant transformation.

The incidence of DNA aneuploidy in this study was higher in the giant CPHN than in the small CPHN. It is known that the risk of malignant melanoma is higher in the giant naevi and it is therefore possible that the presence of aneuploidy indicates a higher risk of malignant change. To date, none of these 17 patients has developed melanoma, so that this is as yet not possible to assess. These patients are part of a long term study of the effects of shaving off the naevi at birth at present in progress at St. Thomas' Hospital and long-term outcome will be assessed subsequently.

When this study was begun, there were no published investigations of the FCM of formalin fixed naevi and the feasibility of this was thus to be assessed; this technique having the obvious benefit of facilitating retrospective study. Our FCM technique has proved to be acceptable for the processing of fixed skin (Newton *et al.*, 1987) provided that enough tissue is available for examination, but technical problems were encountered in this study with melanocytic lesions particularly in terms of a high rejection rate of samples. The majority of the results which could not be interpreted were rejected because of the presence of skewed $G_1$ peaks, of such a size that a near diploid DNA aneuploid clone could not be excluded (Figure 2). The autofluorescence experiment reported provided evidence that autofluorescence of melanin in formalin fixed tissue may be a contributory factor leading to poorly defined DNA histograms in some specimens. It is very unlikely that formalin induced autofluorescence explains all the anomalous results obtained however as skewed $G_1$ peaks are sometimes seen when fresh skin biopsies are run. Other factors linked, for example, to fixation and processing of tissue blocks and their age are probably also involved.

In a recent study of formalin fixed naevi, Von Roenn *et al.* (1986) excluded only 10% of their results. It is difficult to make a meaningful comparison with the present study due to differences in the precise type of lesions studied and variations in tissue processing methods. However, Von Roenn *et al.* (1986) accepted for analysis histograms with skewed $G_1$ peaks and used a slightly higher cut off value of 10% for acceptable CV compared to the 9% used in the present study. Thus it may be that the average quality of results was not markedly better than in the present study where mean CV varied between 4.4 and 5.8% for the various groups. It may be that the use of fresh tissue for DNA studies is preferable where possible. However, despite the technical problems involved, there is good evidence that DNA aneuploidy is more common in lesions with a known risk of progression to malignancy. It will be of considerable interest to see whether the presence of DNA aneuploidy in individual patients is of value in predicting malignant progression. This should become apparent by following the patients included in this and similar studies.

We would like to thank Ms Julie Alder and Mr Michael Stone for their excellent technical assistance. Mr Richard Morris of the Department of Community Medicine. UMDS gave statistical advice.

### References

ALPER, J.C. (1985). Congenital Naevi. The controversy rages on. *Arch. Dermatol.*, **121**, 734.

BARLOGIE, B., DREWINKO, B., SCHUMANN, J. & 5 others (1980). Cellular DNA content as a marker of neoplasia in man. *Am. J. Med.*, **69**, 195.

BORGES, A.F. & LINEBERGER, A.S. (1984). Malignant melanoma without metastases in a giant nevus. *Ann. Plast. Surg.*, **12**, 454.

CLARK JR., W.H., ELDER, D.E., GUERY IV, D., EPSTEIN, M.N., GREENE, M.H. & VAN HORN, M. (1984). A study of tumour progression: the precursor lesions of superficial spreading and nodular melanoma. *Hum. Pathol.*, **15**, 1147.

FRIEDLANDER, M.L., HEDLEY, D.W. & TAYLOR, I.W. (1984). Clinical and biological significance of aneuploidy in human tumours (review). *J. Clin. Pathol.*, **37**, 961.

HEDLEY, D.W., FRIEDLANDER, M.L., TAYLOR, I.W., RUGG, C.A. & MUSGROVE, E.A. (1983). Method for analysis of cellular DNA content of paraffin embedded pathological material using flow cytometry. *J. Histochem. Cytochem.*, **31**, 1333.

NEWTON, J.A., CAMPLEJOHN, R.S. & MCGIBBON, D.H. (1987). The flow cytometry of squamous skin lesions. *Br. J. Dermatol.*, **117**, 169.

PEARSE, A.G.E. (1972). Pigments and pigment precursors. In *Histo-Chemistry, Theoretical and applied*, Pearse, A.G.E. (ed), III, volume 2, ch. 26. Churchill Livingstone: Edinburgh and London.

REED, W.B., BECKER SR., S.W., BECKER JR., S.W. & 0 others (1965). Giant pigmented nevi, melanoma and leptomeningeal melanocytosis: a clinical and histopathological study. *Arch. Dermatol.*, **91**, 100.

RHODES, A.R., SILVERMAN, R.A., HARRIST, T.J. & MELSKI, J.W. (1985). A histologic comparison of congenital and acquired nevomelanocytic nevi. *Arch. Dermatol.*, **121**, 1266.

SONDERGAARD, K., LARSEN, J.K., MOLLER, U., CHRISTENSEN, I.J. & HOU-JENSEN, K. (1983). DNA ploidy characteristics of human malignant melanoma analysed by flow cytometry and compared with histology and clinical course. *Virchows Arch.* (cell pathol.), **42**, 43.

STENZINGER, W., SUTER, L. & SCHUMANN, J. (1984). DNA aneuploidy in congenital melanocytic nevi: suggestive evidence for premalignant change. *J. Invest. Dermatol.*, **82**, 569.

TEODORI, L., CAPURSO, L., CORDELLI, E. & 5 others (1984). Cytometrically determined relative DNA content as an indicator of neoplasia in gastric lesions. *Cytometry*, **5**, 63.

VON ROENN, J.H., KHEIR, S.M., WOLTER, J.M. & COON, J.S. (1986). Significance of DNA abnormalities in primary malignant melanoma and nevi, a retrospective flow cytometric study. *Cancer Res.*, **46**, 3192.